# *The Organon* and Materia Medica Club of the Bay Cities of California

**Published:** 1896-03

**Authors:** W. E. Ledyard

**Affiliations:** Secretary


### *THE ORGANON* AND MATERIA MEDICA CLUB OF THE BAY CITIES OF CALIFORNIA.

The regular semi-monthly meeting was held at the office of Dr. J. M. Selfridge, in Oakland, Friday evening, September 6th, 1895.

Members present: Drs. J. M. Selfridge, M. T. Wilson, A. McNeil, G. J. Auger, S. E. Chapman, George H. Martin, and C. M. Selfridge.

The meeting was called to order at 8.15, by the President, Dr. J. M. Selfridge.

The minutes of the two previous meetings were read, and after a few corrections by Drs. McNeil and C. M. Selfridge, were approved.

Dr. McNeil proposed the name of Dr. M. F. Underwood for membership, stating that the Board of Censors reported favorably upon his name.

The President then appointed Dr. Martin reader of the evening, who commenced at Section 246 of *The Organon.*

### Discussion.

Dr. McNeil—This is explained, and qualified and modified, but I think as it stands it is the only true solution of the problem. In a case of cataract, reported by Dr. Wazcily, which had been examined by an allopathic physician with the ophthalmoscope, as he was going on a journey, I gave one powder of *Sulphur*$^{d}$. I saw him two months later, and as he was improving, I gave no more medicine. In five months he was cured. That one dose allowed the remedy to do its work. It is the secondary action of the drug which annihilates the disease, and should never be interfered with as long as it continues to act. There have been hundreds and thousands of cures made in this way.

Dr. Chapman—Some of my best cures have been made with the single dose. This is the hardest lesson that we, as homœopaths, have to learn.

Dr. J. M. Selfridge—In a case of scirrhus of the breast with a hard, bony feel; sensation of *hot needles* in the chest; stabbing pains, cachexia, etc., I thought *Arsenicum* indicated, and gave one dose of the CM. The patient returned in ten days without much change in the symptoms. I then gave *Sac-lac.* The patient did not return, but wrote me at the first of the month that soon after seeing me the *intolerable burning increased,* which discouraged her very much, and that she had given up the fight. She then went to see a Christian Science woman, and the burning ceased, and that all the indications seemed better. I wrote her that she did not understand the action of homœopathic remedies; that sometimes aggravation follows the use of a remedy, and that she was just as I expected her to be.

Dr. McNeil—I should like to hear from other members on this point. It is a vital question.

Sections 246–248 were then read.

### Discussion.

Dr. Chapman—What is Dr. McNeil's practice in acute diseases as regards the dose?

Dr. McNeil—I give one or two doses in acute diseases. Not, however, in hæmorrhage or *extremis;* but in diphtheria or typhoid, which are both malignant and rapid in their course, I give one or two doses and wait. If I perceive that the drug is the right remedy, I keep on with *Sac-lac.*

Dr. Auger—What would you do in a case of meningitis?

Dr. McNeil—I would do the same thing. This may seem like trifling, but I have seen good results from it in my experience. It is not necessary to repeat the dose in the way spoken of in the note just read.

Dr. Chapman—Hahnemann did not have the extreme high potencies. I think when you use the low potencies it is necessary to repeat the dose.

Dr. McNeil—I never give very high potencies in the beginning of a case. Lippe's rule is to go upwards. W. P. Wesselhoeft's rule is also to go upward. This is what I keep the very high potencies for.

Dr. Chapman—I had a case two or three years ago of membranous croup, in a little girl ten or twelve years of age. I gave *one powder of Bromine*$^{cm}$, and and waited a little while. In fifteen or twenty minutes she had a most violent fit of coughing, and threw off a complete cast of the larynx. This one dose did the work. Last winter the CM potencies cured all my cases, and *I only gave one dose.*

Dr. J. M. Selfridge—I disagree with Dr. McNeil in regard to the repetition of the dose in acute diseases. I have found it best to repeat the dose in such cases. In the case of a little girl with light complexion, suffering from acute dysentery, *Colocynth* was indicated, which I gave in the CC potency, with instruction to give the remedy every two hours until amelioration. I visited her twice a day. In the evening I found her improving, stopped the remedy, and gave *Sac-lac.* She grew worse during the night. I then repeated the remedy and did not cease to continue it, but gave it at longer intervals. She made a rapid recovery. I have tried the single dose in such cases, but almost invariably the patient gets worse; whereas if I continue the remedy, say in pneumonia, there is complete recovery in from four to seven days.

Dr. McNeil—I was led into this method in prescribing in this way: I would be called into a case of cholera infantum, for instance, and would give the indicated remedy saying that if the patient vomits, or when the bowels move, to give another dose; and usually the second dose was not needed. One case, the Rev. Dr. —— sent for me. He was suffering with cholera morbus; violent paroxysms; *fainted when bowels moved.* It was a clear case for *Bryonia.* I gave the CC potency, and he did not have another movement for twenty-four hours. In croup I give one dose, and say that if another paroxysm comes on to give it again; but it does not come on. My own little girl woke out of sleep with croup, aggravated by sleep at 2.30 A. M. I gave her one dose of *Lachesis*, and she slept until morning.

Dr. Wilson—My practice is to give the medicine, repeating the dose until I get some action, and then cease. It is hard for us to give one dose and then quit, especially in acute troubles.

I believe in commencing with the low potencies and going higher. Guernsey used to teach this, and Lippe the same.

Dr. Chapman—Suppose in a case of dysentery we give the CC potency, and order it repeated after there is an operation. Very often there is not more than one or two doses required. I have also done as Dr. Selfridge does.

Dr. Auger—A case of mine—cerebral meningitis. Four or five allopaths had attended and given up. Child four years old; unconscious; pupils dilated; spasms; vomiting; opisthotonos. I gave *Belladonna* $^{cc}$, to be repeated every two hours. When I went at noon the vomiting was less frequent, and had but two spasmodic attacks. I ordered the remedy still repeated. In the evening there was no vomiting; no spasms; was much better, but complained of head. I continued the remedy at the same intervals during the night. In the morning was a little better; had slept five, ten, and fifteen minutes at first; now slept an hour at a time quietly. I still continued the remedy, but every *three* hours. I debated whether I should discontinue the remedy during the night, but decided that I would not stop it.

Dr. McNeil—I mix the remedy in water, and say if the spasm, etc., comes on to give the remedy again.

Dr. Auger—How about the headache in my case?

Dr. McNeil—That was a good sign. In typhoid, at my first visit, I give the remedy, and at my second visit *Sac-lac.* All these cases of typhoid get well in five days. I believe that every repetition of the dose interrupts the action of the drug, and thereby does harm.

Dr. Chapman—It is like eating; after you have eaten enough, if you eat any more you eat too much.

Dr. McNeil—There is danger in repeating the dose. Where the first dose will cure, the second will put the case back where you started from.

Section 249 was now read.

### Discussion.

Dr. McNeil—Here is a very valuable rule which we should remember. We should know whether we have an aggravation or the regular course of the disease. If you give a drug, and *new symptoms* are produced, *that aggravation is harmful.* In such a case we have good reason to assume that the new symptoms are the result of the drug.

Sections 250 and 251.

Upon motion of Dr. Martin, seconded by Dr. Chapman, the meeting then adjourned to meet again the third Friday in September at the office of Dr. M. T. Wilson, 824 Ellis Street, San Francisco, when the reading of *The Organon* would be commenced at Section 252.

W. E. LEDYARD, M. D., *Secretary.*

Reported by Eleanor F. Martin, M. D.

---

